# Comparison of Tooth- and Bone-Borne Appliances on the Stress Distributions and Displacement Patterns in the Facial Skeleton in Surgically Assisted Rapid Maxillary Expansion—A Finite Element Analysis (FEA) Study

**DOI:** 10.3390/ma14051152

**Published:** 2021-03-01

**Authors:** Rafał Nowak, Anna Olejnik, Hanna Gerber, Roman Frątczak, Ewa Zawiślak

**Affiliations:** 1 Department of Maxillofacial Surgery, Wroclaw Medical University, Borowska 213, 50-556 Wrocław, Poland; anna.olejnik@umed.wroc.pl (A.O.); hanna.gerber@umed.wroc.pl (H.G.); ewa.zawislak@umed.wroc.pl (E.Z.); 2 NOBO Solutions S.A., Al. Kasztanowa 3A-5, 53-125 Wrocław, Poland; roman.fratczak@nobosolutions.com

**Keywords:** SARME, transpalatal distraction, finite element analysis, maxillary constriction, maxillary expansion, orthognathic surgery

## Abstract

The aim of this study was to compare the reduced stresses according to Huber’s hypothesis and the displacement pattern in the region of the facial skeleton using a tooth- or bone-borne appliance in surgically assisted rapid maxillary expansion (SARME). In the current literature, the lack of updated reports about biomechanical effects in bone-borne appliances used in SARME is noticeable. Finite element analysis (FEA) was used for this study. Six facial skeleton models were created, five with various variants of osteotomy and one without osteotomy. Two different appliances for maxillary expansion were used for each model. The three-dimensional (3D) model of the facial skeleton was created on the basis of spiral computed tomography (CT) scans of a 32-year-old patient with maxillary constriction. The finite element model was built using ANSYS 15.0 software, in which the computations were carried out. Stress distributions and displacement values along the 3D axes were found for each osteotomy variant with the expansion of the tooth- and the bone-borne devices at a level of 0.5 mm. The investigation showed that in the case of a full osteotomy of the maxilla, as described by Bell and Epker in 1976, the method of fixing the appliance for maxillary expansion had no impact on the distribution of the reduced stresses according to Huber’s hypothesis in the facial skeleton. In the case of the bone-borne appliance, the load on the teeth, which may lead to periodontal and orthodontic complications, was eliminated. In the case of a full osteotomy of the maxilla, displacements in the buccolingual direction for all the variables of the bone-borne appliance were slightly bigger than for the tooth-borne appliance.

## 1. Introduction

Maxillary transverse extension is the method of choice in the treatment of disproportions in the transverse dimensions of both the maxilla and the mandible, which are, among others, distinguished by crossbite [1]. During maxillary expansion, the maxillary and palatine bones are separated in the palatal suture, which increases the upper arch width. After their separation, the goal of treatment is to increase the bone base of the maxilla by providing stimulation to form new bone. The optimal anterior and posterior widths of the upper arch have a substantial impact on stable occlusion after the orthodontic treatment or the orthodontic and surgical team treatment is completed [2]. The causes of maxillary constriction include genetic and environmental factors, as well as dysfunctions and parafunctions, namely, mouth breathing or thumb sucking [3]. According to the “functional matrix hypothesis” formulated by Moss in the 1960s, the proper function and growth of the facial skeleton are determined by proper breathing, swallowing and chewing [4,5]. The absence of appropriate functional stimuli results in the disordered morphology of the facial skeleton and the occurrence of malocclusion.

The anatomy and architecture of the midface are of fundamental importance in maxillary expansion therapy [6,7]. There are various expansion protocols: slow maxillary expansion (SME), semirapid maxillary expansion (SRME), rapid maxillary expansion (RME) and surgically assisted maxillary expansion [8,9]. With age, the elasticity of bone and the ability to treat disorders with orthodontic and orthopaedic methods decreases. As a result, surgical procedures are required [10,11]. It was originally believed that ossification of the palatal suture blocked orthodontic expansion of the maxilla in adult patients. However, Schlegel et al. found that suture ossification occurred in only half of the study group who were over 23 years of age [12].

Further studies on the architecture of the facial skeleton and separation of the palatal suture showed that the points of increased bone resistance in the midface made it impossible to perform maxillary expansion in adult patients [13,14]. The sites with increased bone resistance are located in the so-called Sicher’s pillars, i.e., the canine, zygomatic and pterygoid process pillars, and in the midpalatal suture [15]. The research studies conducted by Lines, as well as Bell and Epker, shed light on SARME (surgically assisted rapid maxillary expansion) therapy and aimed at increasing its transverse dimensions [14,15,16]. The authors argued that in the facial skeleton, there are more sites of bone resistance, apart from the palatal suture in adult patients undergoing maxillary expansion [17]. The sites of increased resistance within the midface region turned out to be the apertura piriformis (anterior resistance point), the zygomaticoalveolar crest (lateral resistance point) and the pterygopalatine suture (posterior resistance point) [1]. A surgical incision into the bone resistance sites in the facial skeleton described by Sicher enables performing an expansion of the maxilla, and thus minimising the stresses in other bones of the skull and the cranial base [10,18,19,20]. At the present time, there is no settled conclusion regarding the type of optimum osteotomy of the midface in surgically assisted rapid maxillary expansion in the literature [21,22].

Another important aspect in surgically assisted rapid maxillary expansion is the type of force-generating device used for expanding the maxilla.

In practice, devices are fixed onto teeth, directly to the bone or hybrid fixation appliances are used [1,17,23]. Tooth-borne orthodontic appliances indirectly apply forces to the facial skeleton by exerting their effect through the teeth. The direct fixation of the device to the bone allows for ignoring the effects exerted by the teeth. In MARPE (microimplant-assisted rapid palatal expansion) therapy, the teeth and the palatal process of the maxilla are used for fixing the device. MARPE is a combination of the two types of device fixation mentioned above [24,25]. Each type of expander has its supporters and opponents. However, the aspect of loading the patient’s teeth with the tooth-borne appliance and its negative effects on the periodontium are widely discussed in the literature [21,25,26]. Another undesirable effect of the tooth-borne appliance is a significant dentoalveolar effect, which may have an influence on the lasting effects of orthodontic treatment [3,7].

By taking into account both the surgical and orthodontic aspects, this analysis aimed at finding an optimal biomechanical solution. In order to facilitate the selection of the best method of treatment in surgically assisted rapid maxillary expansion therapy, five surgical variants and two types of expanders were modelled.

## 2. Material and Methods

### 2.1. Design of the Facial Skeleton Model for the Finite Element Analysis

**Figure 1:**
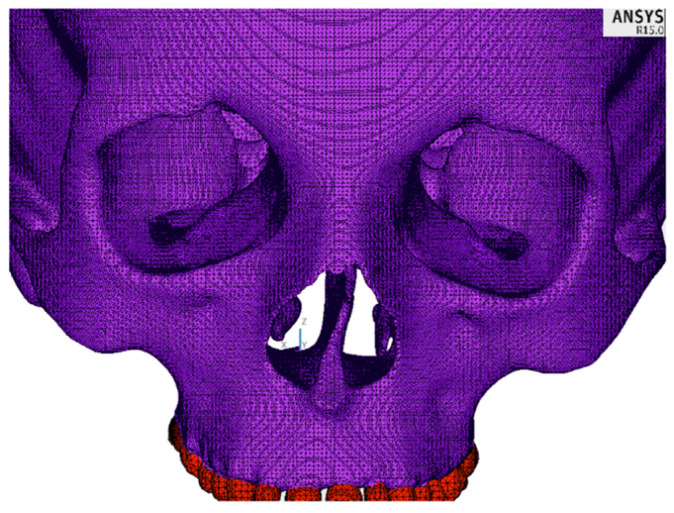
A fragment of the finite element mesh.

The three-dimensional (3D) model of the skull was created based on spiral computed tomography (CT) scans of a healthy 32-year-old female patient with maxillary constriction. DICOM (Digital Imaging and Communications in Medicine) files were imported into the Slicer3D program (Slicer 4.10.2^®^, BWH, Boston, MA, USA), in which the facial bones were separated. The separation was made by marking the attenuation thresholds for the bone structure. CT scan voxels with attenuation in that scope formed a volumetric model of the patient’s facial skeleton. Subsequent transformation in the form of a surface mesh in the stereolithography (.stl) format was carried out in the Blender software (Blender 2.82a^®^, Blender Fundation, Amsterdam, The Nederland). The STL format represents a three-dimensional surface in triangular facets. In this way, a high-quality surface mesh was produced. The surface mesh was exported in the STL format to the GMSH program (GMSH 4.1.^®^, GMSH, Brussels, Belgium), where a resulting volumetric mesh of 10 nodal tetrahedrons of the second order was created. In order to generate a dense mesh of higher-order finite elements, the mesh was imported into the ANSYS 15.0 software (Swanson Analysis System, Cannonsburg, PA, USA). ANSYS is a finite element analysis (FEA) software tool for structural analysis in a simulated environment. In ANSYS 15.0, the mechanical properties of the materials in the models were assigned, as shown in Table 1, and an analysis using the finite element method was made [24,27,28,29]. As a result of the dense discretisation, a dense mesh of higher-order finite elements was created, consisting of over 1.8 million elements, with an element edge length of 1–2 mm (Figure 1).

### 2.2. Design of the Bone- and Tooth-Borne Appliances

**Figure 2:**
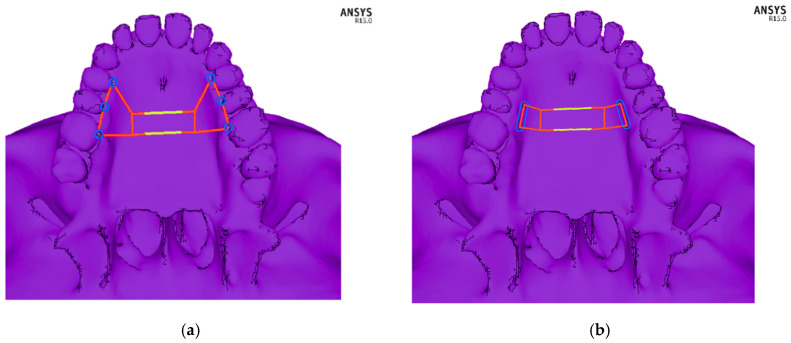
Schematic picture of the designed orthodontic devices: (**a**) fixed to the bone—bone-borne type, and (**b**) fixed onto teeth—tooth-borne type. The module that was expanded by 0.5 mm is marked in yellow.

**Figure 3:**
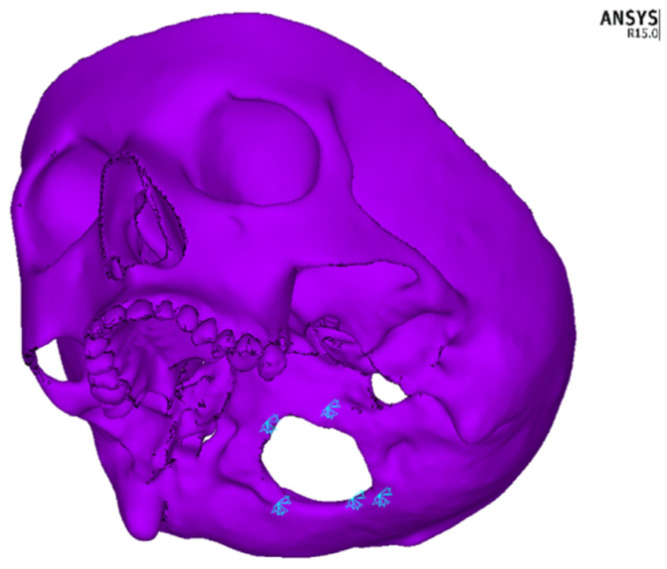
Fixation points of the model in the foramen magnum region.

Two variants of the appliance with different methods of fixing were modelled using beam elements. For the arms of the device, a 3.5 mm diameter wire was established, and for the central part, a 3 mm diameter wire was established. In the first variant, the implant was fixed directly to the maxillary bone (bone-borne type) between teeth 4 and 5, as well as teeth 5 and 6. In the second variant, the implant was fixed onto teeth 4, 5 and 6 of the maxilla (tooth-borne type). The expansion of the central part of the device by 0.5 mm was modelled by assigning to its central part of 9.62 mm in length an artificial coefficient of thermal expansion with a value of 5.197 × 10^−4^ and raising its temperature by 100 °C. In clinical cases, the expansion of the device occurs by turning the screw at the human body temperature. The views of both appliances together with the marked fixation points on the hard palate and teeth, including the part which was expanded, are presented in Figure 2. On the model of the facial skeleton, there were five points of fixation around the foramen magnum, as shown in Figure 3.

### 2.3. Models of Osteotomy of the Midface

**Figure 4:**
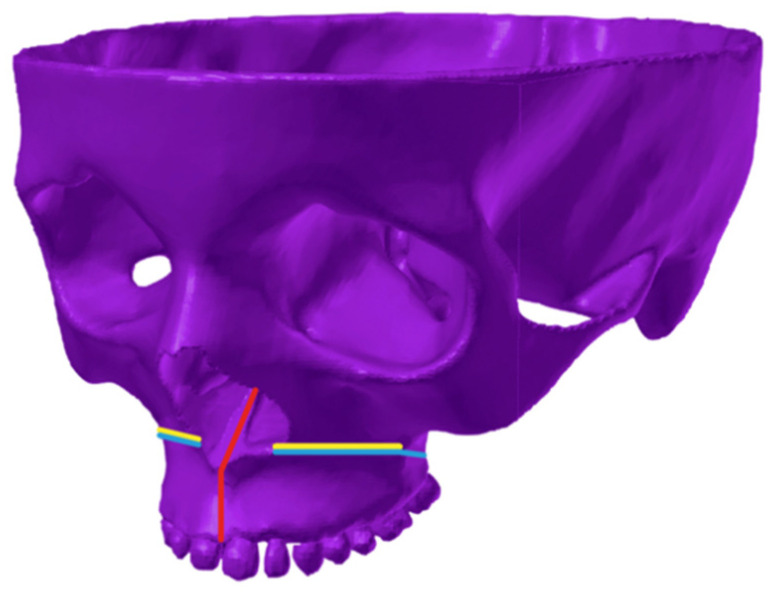
Schematic representation of the osteotomy line on the 3D finite element model of the facial skeleton. Red colour—palatal suture osteotomy; yellow colour—Fort I osteotomy without a PMJ separation; blue colour—total Le Fort I osteotomy.

**Figure 5:**
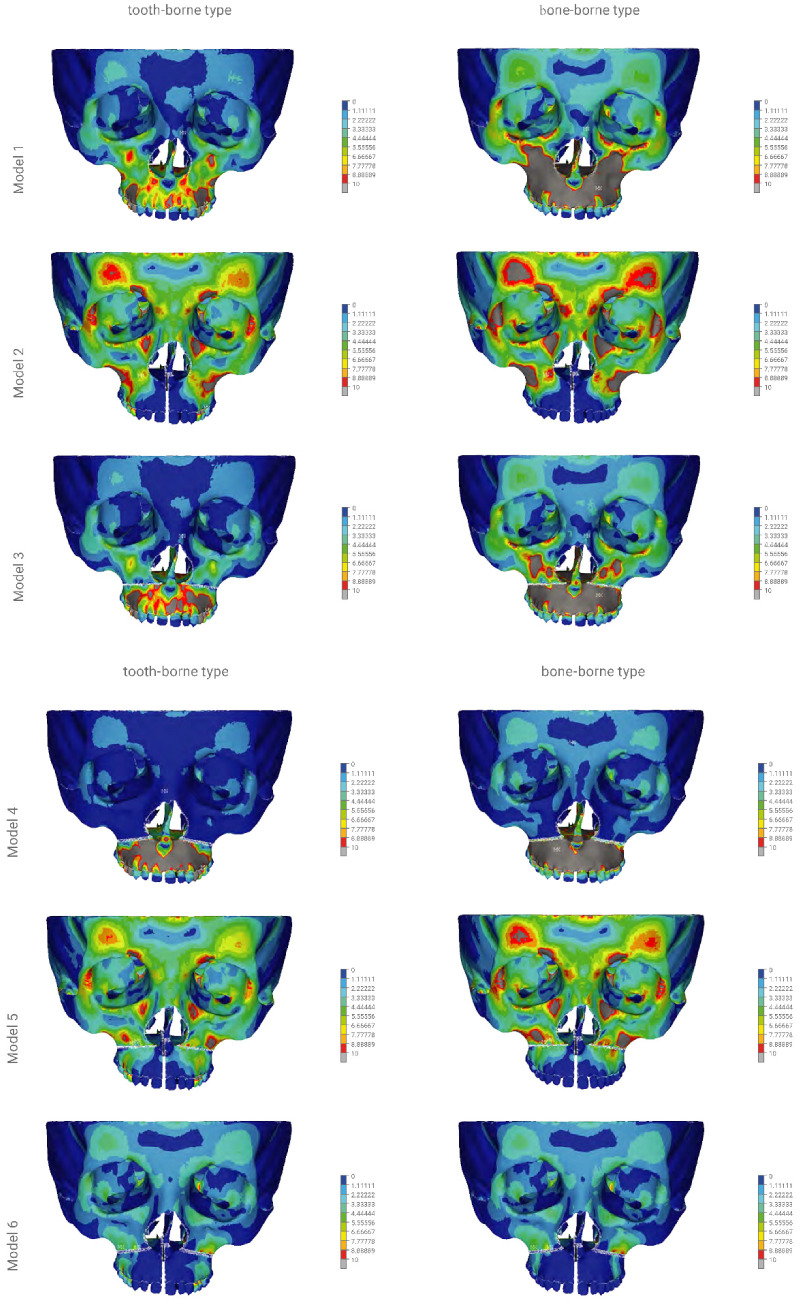
The finite element model showing the front view of the distribution of the stresses (scale 0–10 MPa) according to Huber throughout the craniofacial skeleton as a result of palatal expansion using five different surgical procedures, and without an osteotomy. Model 1—without an osteotomy; model 2—sagittal osteotomy; model 3—transversal osteotomy modo Le Fort I without a PMJ separation; model 4—transversal osteotomy modo Le Fort I with a PMJ separation; model 5—sagittal osteotomy with a transversal osteotomy modo Le Fort I without a PMJ separation; model 6 (the model used for finite element analysis)—sagittal osteotomy with a transversal osteotomy modo Le Fort I with a PMJ separation.

**Figure 6:**
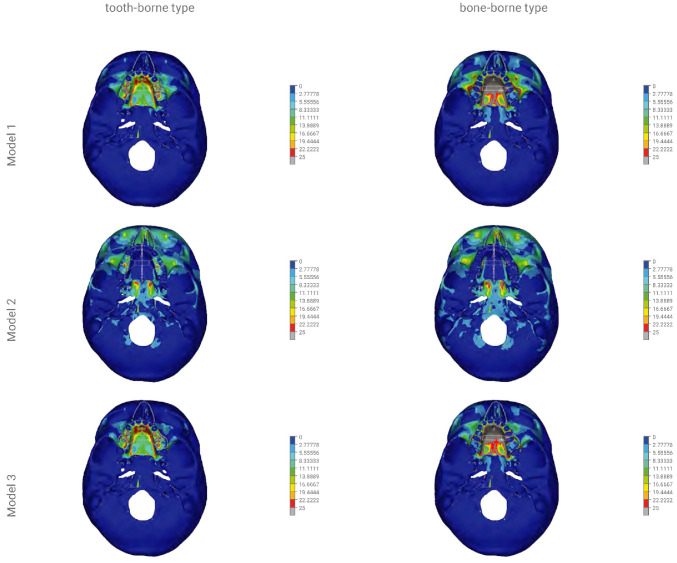

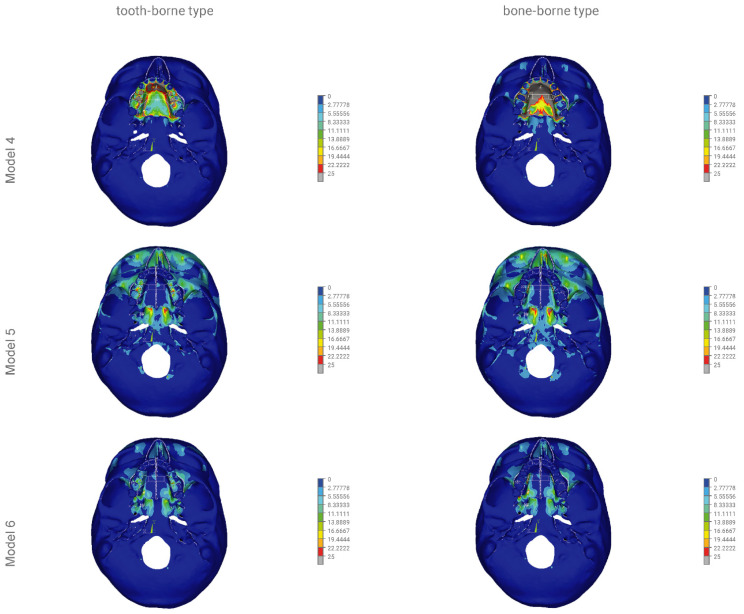
The finite element model showing the bottom view of the distribution of stresses (scale 0–25 MPa) according to Huber throughout the craniofacial skeleton as a result of palatal expansion using five different surgical procedures, and without an osteotomy. Model 1—without an osteotomy; model 2—sagittal osteotomy; model 3—transversal osteotomy modo Le Fort I without a PMJ separation; model 4—transversal osteotomy modo Le Fort I with a PMJ separation; model 5—sagittal osteotomy with a transversal osteotomy modo Le Fort I without a PMJ separation; model 6 (the model used for finite element analysis)—sagittal osteotomy with a transversal osteotomy modo Le Fort I with a PMJ separation.

By using dense discretisation, the facial skeleton models were created with five variants of osteotomy of the facial skeleton and one without surgical intervention. Each model was analysed using the tooth-borne device and the bone-borne device with a central module expansion of 0.5 mm. In Figure 4, the course of the osteotomy line is shown schematically. Three colours were used to mark various types of osteotomies: a red one in case of only an osteotomy in the palatal suture, yellow for a Le Fort I osteotomy without a separation from the pterygoid processes of the sphenoid bone and blue for a total separation of the maxilla. In total, 12 numerical models were analysed: model 1—a variant without surgical intervention (tooth-borne, bone-borne), model 2—sagittal osteotomy in the palatal suture region (tooth-borne, bone-borne), model 3—osteotomy according to the Le Fort I line without a separation in the pterygomaxillary junction (PMJ; tooth-borne, bone-borne), model 4—osteotomy according to the Le Fort I line with a separation in the PMJ (tooth-borne, bone-borne), model 5—sagittal osteotomy combined with an osteotomy according to the Le Fort I line without a separation in the PMJ (tooth-borne, bone-borne) and model 6—full osteotomy of the maxilla according to the Le Fort I line with a separation in the PMJ (tooth-borne, bone-borne). Table 2 provides a detailed description of the range of the modelled osteotomies and the order in which the maps of the reduced stresses according to Huber are presented in Figure 5 and Figure 6.

The reduced stresses according to Huber’s hypothesis were determined for the selected anatomical structures of the facial skeleton for the 12 models of the skull. In models 1a to 6a, the tooth-borne appliance was used, whereas in models 1b to 6b, the bone-borne appliance was used. Furthermore, coloured contour stripes show the distribution of the reduced stresses at a particular site in the front and bottom views in Figure 5 and Figure 6, as in the order shown in Table 2.

The displacements in millimetres along the *X*-, *Y*- and *Z*-axes were determined for selected anatomical structures of the facial skeleton for the tooth- and bone-borne appliances for the 12 numerical models of the skull.

The displacement along the *X*-axis correlated with a shift in the buccolingual direction. Positive values describe displacements in the buccal direction and negative values describe displacements in the lingual direction.

The displacement along the *Y*-axis correlated with a shift in the anterior–posterior direction. Positive values describe displacements in the anterior direction and negative values describe displacements in the posterior direction.

The displacement along the Z-axis correlated with the shift in the superior–inferior direction. Positive values describe displacements in the superior direction and negative values describe displacements in the inferior direction.

## 3. Results

### 3.1. Stresses Reduced According to Huber’s Hypothesis

Figure 5 and Figure 6 show the distribution of the reduced stresses according to Huber’s hypothesis for activation at a level of 0.5 mm for the bone- and tooth-borne appliances for the 12 numerical models of the skull in the front and bottom views. Moreover, Table 3 presents the distribution of the stresses for selected points of the facial skeleton for the 12 variants under study.

In model 1, the activation of the tooth-borne device over the range of 0.5 mm resulted in the distribution of reduced stresses according to Huber to 3.3 MPa within the orbital region and the squamous part of the frontal bone. The highest values of >10 MPa were found in the region of the maxillary alveolar process at the premolars, on the anterior wall of the maxillary sinuses and in the hard palate. The activation of the bone-borne device over the range of 0.5 mm in model 1 showed the distribution of the reduced stresses according to Huber to 5.5 MPa within the orbital region and the squamous part of the frontal bone. The highest values of >10 MPa were found within the proximity of the entire region of the body of both maxillary bones. The values of >25 MPa were found in the entire region of the hard palate.

In model 2, the activation of the tooth-borne device over the range of 0.5 mm resulted in an increase in the stress reduction according to Huber to 10 MPa within the orbital region and the squamous part of the frontal bone. In the region of the maxillary alveolar process in the anterior part, the stresses decreased to 1.1 MPa, and similar values were found in the anterior and posterior regions of the hard palate. The activation of the bone-borne device in the range of 0.5 mm in model 2 showed an increase in the stress reduction according to Huber to >10 MPa within the orbital region and the squamous part of the frontal bone. In the region of the maxillary alveolar process in the anterior part, the stresses decreased to 1.1 MPa, and similar values were found in the anterior and posterior regions of the hard palate.

In model 3, the activation of the tooth-borne device over the range of 0.5 mm resulted in the distribution of reduced stresses according to Huber to 3.3 MPa within the orbital region and the squamous part of the frontal bone. The highest values of >10 MPa were found in the region of the maxillary alveolar process in the proximity of premolars. The values of up to 25 MPa in the anterior region and up to 13.8 MPa in the posterior region were found in the hard palate. The activation of the bone-borne device over the range of 0.5 mm in model 3 showed an increase in the stress reduction according to Huber to 6.6 MPa within the orbital region and the squamous part of the frontal bone. In the entire region of the maxillary alveolar process and on the anterior wall of the maxillary sinus, the stress values increased and were >10 MPa. In the anterior and posterior regions of the hard palate, they also increased and were >25 MPa.

In model 4, the activation of the tooth-borne device over the range of 0.5 mm resulted in the distribution of reduced stresses according to Huber to 2.2 MPa within the orbital region and the squamous part of the frontal bone. The highest values of >10 MPa were found in the entire region of the maxillary alveolar process. Values of up to 25 MPa in the anterior region and up to 13.8 MPa in the posterior region were found in the hard palate. The activation of the bone-borne device over the range of 0.5 mm in model 4 showed an increase in the stress reduction according to Huber to 3.3 MPa within the orbital region and the squamous part of the frontal bone. In the entire region of the maxillary alveolar process, the stress values increased and were >10 MPa. In the anterior and posterior regions of the hard palate, they also increased and were >25 MPa.

In model 5, the activation of the tooth-borne device in the range of 0.5 mm resulted in the distribution of reduced stresses according to Huber to 10.0 MPa within the orbital region and the squamous part of the frontal bone. In the region of the maxillary alveolar process in the anterior part, the stresses were decreased to 1.1 MPa, and similar values were found in the anterior and posterior regions of the hard palate. The activation of the bone-borne device over the range of 0.5 mm in model 5 showed an increase in the stress reduction according to Huber to 10 MPa within the orbital region and the squamous part of the frontal bone, as well as in the region of the zygomaticoalveolar crest. In the region of the maxillary alveolar process in the anterior part, the stresses were decreased to 1.1 MPa, and similar values were found in the anterior and posterior regions of the hard palate.

In model 6, the activation of the tooth-borne device in the range of 0.5 mm resulted in the distribution of reduced stresses according to Huber to 3.3 MPa within the orbital region and the squamous part of the frontal bone. In the region of the maxillary alveolar process in the anterior part, the stresses decreased to 1.1 MPa, and similar values were found in the anterior and posterior regions of the hard palate. The activation of the bone-borne device in the range of 0.5 mm in model 6 showed the distribution of reduced stresses according to Huber to 4.4 MPa within the orbital region and the squamous part of the frontal bone. In the region of the maxillary alveolar process in the anterior part, the stresses decreased to 1.1 MPa, and similar values were found in the anterior and posterior regions of the hard palate.

### 3.2. Displacements of Selected Facial Skeleton Structures along the X-, Y- and Z-Axes

#### 3.2.1. Displacements of Selected Facial Skeleton Structures along the *X*-Axis

In model 1 with the tooth-borne appliance, the maximum displacement along the *X*-axis was 0.04 mm. In model 2, it fluctuated from 0.31 to 0.22 mm; in model 3, it reached a maximum of 0.04 mm; in model 4, it fluctuated from 0.04 to 0.13 mm; in model 5, from 0.22 to 0.31 mm; in model 6, from 0.22 to 0.4 mm. The maximum shift along the *X*-axis was found at the mesial incisal angle of the central maxillary incisor (0.4 mm) in model 6 of the facial skeleton. In model 1 with the bone-borne appliance, the shift along the *X*-axis ranged from 0.04 to 0.13 mm. In model 2, it ranged from 0.22 to 0.4 mm; in model 3, from 0.04 to 0.13 mm; in model 4, from 0.04 to 0.22 mm; in model 5, from 0.22 to 0.4 mm; in model 6, from 0.31 to >0.4 mm. The maximum shift in the buccal direction (>0.4 mm) was found at the mesial incisal angle of the central maxillary incisor in model 6 of the facial skeleton for the bone-borne appliance.

#### 3.2.2. Displacements of Selected Facial Skeleton Structures along the *Y*-Axis

In model 1 with the tooth-borne appliance, the shift along the *Y*-axis ranged from −0.05 to 0.01 mm. In model 2, it ranged from 0.03 to 0.05 mm; in model 3, from −0.5 to 0.03 mm; in model 4, from −0.05 to 0.05 mm; in model 5, from 0.03 to 0.05 mm; in model 6, from −0.01 to 0.07 mm. The maximum shift in the anterior direction (0.07 mm) was found at the mesial incisal angle of the central maxillary incisor in model 6 of the facial skeleton. For the bone-borne appliance, the displacement along the *X*-axis in model 1 fluctuated from >−0.1 to 0.01 mm. In model 2, it ranged from 0.03 to 0.07 mm; in model 3, from >−0.1 to 0.01 mm; in model 4, from >−0.1 to 0.07 mm; in model 5, from 0.03 to 0.07 mm; in model 6, from −0.03 to 0.05 mm. The maximum shift in the posterior direction was found at the mesial incisal angle of the central maxillary incisor (>−0.1 mm) in models 1, 3 and 4 of the facial skeleton for the bone-borne appliance.

#### 3.2.3. Displacements of Selected Facial Skeleton Structures along the *Z*-Axis

In model 1 with the tooth-borne appliance, the shift along the *Z*-axis ranged from −0.03 to 0.03 mm. In model 2, it fluctuated from 0.01 to 0.07 mm; in model 3, from −0.3 to 0.03 mm; in model 4, from −0.03 to −0.07 mm; in model 5, from 0.05 to 0.07 mm; in model 6, from −0.05 to 0.07 mm. The maximum shift in the anterior direction (0.07 mm) was found at the mesial incisal angle of the central maxillary incisor in model 6 of the facial skeleton. For the bone-borne appliance, the displacement along the *X*-axis in model 1 ranged from −0.05 to 0.1 mm. In model 2, it fluctuated from 0.05 to 0.03 mm; in model 3, from −0.5 to 0.03 mm in model 4, from −0.05 to 0.05 mm; in model 5, from 0.03 to 0.05 mm; in model 6, from −0.01 to 0.07 mm. The maximum shift in the superior direction (>0.1 mm) was found on the posterolateral surface of the maxilla in model 4 of the facial skeleton.

Table 4 represents detailed displacement values (mm) along the *X*-, *Y*- and *Z*-axes at selected points.

## 4. Discussion

Surgically assisted rapid maxillary expansion allows for obtaining optimum widths of the upper dental arch in patients whose bone growth is complete [1,3,6,13,17]. According to various authors, the ages of 14–18 years is the restriction for orthodontic treatment [16,17,21]. Handelman et al. show an alternative view [30]. The authors report that adult patients with maxillary constriction are treated successfully without surgical procedures.

Mommaerts, who developed the method of surgically assisted maxillary expansion with a bone-borne appliance, suggests performing an osteotomy of the anterior, lateral and medial maxillary buttresses [1]. According to the authors, such incision conditions correct a separation in the fragments of the maxilla and its expansion during active distraction therapy. A similar opinion is presented by Reinbecheret et al. [31]. The researchers conducted a study on a group of 25 patients treated with SARME using the same osteotomy as in Mommaerts, also without nasal septum separation. The results of the analysis show that it is not necessary to separate the nasal septum when a surgical procedure of maxillary expansion at 5–11 mm is performed [31]. Nasal septum separation is required in asymmetric expansions [1].

There is currently no opinion in the literature regarding the type of osteotomy that is recommended for the midface in surgically assisted rapid maxillary expansion [1,17,21,22].

Long-term observations of lasting treatment effects may indicate the need for incomplete separation of the maxilla from the facial skeleton, along with a sagittal osteotomy of the palatal suture [32,33]. Seeberger et al. report that an incision made in the maxilla according to the protocol by Bell and Epker from 1976 without separation of the pterygopalatine suture in SARME therapy results in a nonrecurrent defect throughout observations made after 5 years following surgery [32].

Studies using the finite element method (FEM) indicate the need for performing a full sagittal and transversal osteotomy of the maxilla to minimise the risk of stresses in the facial skeleton and the neurocranium, as well as possible, uncontrolled fractures [7,11,20]. Laningan et al. describe rare ophthalmic complications after a Le Fort I osteotomy as complications from orthognathic surgery. Complications include impaired visual acuity, extraocular muscle dysfunction, keratitis and lacrimal duct injury [33]. The abovementioned problems may result from an indirect injury to the orbital structures caused by traction or compression, especially when the maxilla is separated from the pterygoid process of the sphenoid bone [33,34]. FEAs performed in maxillary expansion therapy show that the highest stresses, including the risk of fracture, occur in the region of the body of the sphenoid bone. This may result in the formation of a carotid cavernous fistula, injury to the internal carotid artery and paralysis of the cranial oculomotor nerves (III, IV, VI), which leads to ophthalmoplegia [7,11,20].

In practice, devices fixed onto teeth, directly to the bone or hybrid fixation appliances are used. Each type of expander has its supporters and opponents [35,36].

Finite element analysis enables simulations and analysis of a mathematical model for a given process or status of a physical system. It is commonly used by researchers to analyse stresses and strains in complex mechanical systems [37]. Finite element analysis is a good method for assessing modelled variants of the treatment of maxillary constriction in adult patients who require surgical procedures [7,11,20].

Reviewing the literature, there has been a huge technological shift in maxillofacial surgery in the recent past. Applications of FEM, computer-assisted surgical planning, 3D printing technology or intraoperative navigation can potentially improve the efficiency and predictability of the surgical treatment [38,39].

The analysis executed by the authors indicates significant differences in the distribution of the reduced stresses according to Huber, as well as displacement patterns for the modelled variants of osteotomies and the two types of appliances used for maxillary expansion.

In this study, the bone-borne appliance generated the largest increase in reduced stresses according to Huber, both in terms of the covered area of the facial skeleton and their values (>10 MPa) in models 1, 3 and 4. In model 1 without an osteotomy, the stresses were >10 MPa within the entire region of the body of the maxilla and its processes, and >25 MPa in the hard palate, excluding the maxillary teeth. In models 3 and 4, the stresses were >10 MPa within the entire region of the body of the maxilla and its processes below the osteotomy line and >25 MPa in the entire hard palate, including the maxillary teeth. As a result of the PMJ separation in model 3, the stresses on the anterior wall of the maxillary sinus increased to >10 MPa.

The tooth-borne appliance did not generate such an extensive and massive increase in the stresses in models 1 and 3 in the region of the body of the maxilla and all maxillary processes. In model 4, the distribution and level of stresses were similar for the tooth- and bone-borne appliances, excluding the teeth and the posterior region of the hard palate, to the advantage of the tooth-borne appliance.

To sum up, the absence of an osteotomy of the palatal suture when the bone-borne appliance was fixed significantly increased the stresses on the entirety of the maxilla, with a maximum displacement of 0.22 mm along the *X*-axis in the buccolingual direction at the level of buccal cusps of upper teeth 5 (model 4).

An osteotomy of the palatal suture only (model 2) using both the tooth- and bone-borne appliances resulted in similar distributions of stresses in the facial skeleton. The maximum values of the reduced stresses according to Huber (>10 MPa) were found in the orbital region and the squamous part of the frontal bone. Our analysis revealed that the type of expander used for isolated palatal suture osteotomy had no impact on the distribution of the reduced stresses in the facial skeleton. For all variables, the shifts in the buccolingual direction (*X*-axis) were slightly bigger for the bone-borne appliance. The absence of references regarding this issue in the literature may result from the absence of isolated sagittal osteotomy in surgically assisted maxillary expansion.

The distribution of reduced stresses was similar for the tooth- and bone-borne appliances in models 5 and 6 in the facial skeleton. Separation in the pterygopalatine suture had a positive effect on the reduction of stresses in the orbital region, the squamous part of the frontal bone and the anterior wall of the maxilla and teeth on which the tooth-borne appliance was fixed, up to the maximum value of 5.5 MPa (model 6).

Our analysis revealed that the reduction of stresses on teeth onto which the appliance was fixed requires an osteotomy of the pterygopalatine suture in SARME therapy.

An osteotomy of the pterygopalatine suture in surgically assisted rapid maxillary expansion therapy is also a subject of discussion [17,22]. Sangasari et al. found no statistically significant difference in the treatment results for the separation of the maxilla from the pterygoid process of the sphenoid bone [22]. Killiç et al. and Laudemann et al. present an alternative view [34,40]. The authors indicate that the separation of the pterygopalatine suture significantly influences the expansion of the maxilla and the rotation of the osteotomy fragments. Separation of the maxilla from the pterygoid processes of the sphenoid bone extends the chance of surgical complexity, for instance, a descending palatal artery or a pterygoplexus haemorrhage, and as a result, osteonecrosis of the maxilla [17,22].

The tooth fixation of the appliance generated loads on teeth in all modelled variants. The lowest values of the reduced stresses on the teeth were found in the model with a full osteotomy (model 6).

There is an extensive literature on the negative impact of loading teeth onto which the appliance is fixed [1,17,25,26]. The undesirable effects include compression and bone loss, tooth mobility, deflection and extrusion, bone dehiscence and gingival recession [41,42,43]. The abovementioned problems are often complicated by hygienisation difficulties that are experienced when such an appliance is used. Transpalatal distractors are defined as hygienic devices that are easy to use [19,21].

The shifts in the buccolingual direction (*X*-axis) were bigger for the bone-borne appliance for all the variables under study in models 5 and 6, excluding the measurement on the cusp of the tooth onto which the appliance was fixed (Table 4). Displacements that were almost twice as big for the bone-borne fixation along the *X*-axis were found in model 6 at the lowest point of the apertura piriformis (0.22 vs. 0.4 mm—tooth-borne vs. bone-borne). This reveals that the bone-borne appliance that was used for maxillary expansion exerted a skeletal effect, not a dental effect.

The methods using tooth-borne appliances are associated with the risk of defect recurrence and the need to carry out treatment with an overcorrected expansion [1,44,45]. It is probably caused by the significant dental effect of expansion and not the dominant one exerted on the facial skeleton.

Slight differences in displacements in the *Y*- and *Z*-directions for the tooth- and bone-borne fixations are not the subject of our analysis and therefore will not be discussed.

## 5. Conclusions

The analysis executed by the present authors revealed the following:

- An absence of surgical support for maxillary expansion significantly increased the stress reduction according to Huber’s hypothesis in the entire facial skeleton in patients whose bone growth is complete, without affecting displacements in the buccolingual dimension (*X*-axis).
- Higher levels of stresses and covered areas of the facial skeleton were found for the bone-borne appliance, especially in the model without an osteotomy and in models without an incision in the palatal suture.
- Only in the case of a complete separation of the maxilla at all its junctions, as well as an osteotomy of the palatal suture, the type of expander (tooth-borne vs. bone-borne) had no significant effect on the distribution of the reduced stresses in the facial skeleton.
- Compared to the tooth-borne appliance, the bone-borne expander allowed for a reduced load on the periodontium in all the modelled osteotomy variants and in the model without surgical intervention.
- Compared to the tooth-borne appliance, the bone-borne appliance exerted a bone expansion effect on the models with a full osteotomy, as confirmed by the displacements along the *X*-axis for selected variables.
- A transpalatal distraction may be an effective method of treating maxillary constriction in adult patients in the case of total separation of the maxilla, combined with palatal suture separation.

## Figures and Tables

**Table 1:** The mechanical properties of the components of the finite element model that was constructed for the present study [27,28,29].

Variable	Young’s Modulus (MPa)	Poisson’s Ratio
Compact bone	13,700	0.26
Cancellous bone	1370	0.3
Enamel	80,000	0.26
Dentin	20,000	0.15
Stainless steel	200,000	0.3

**Table 2:** A detailed course of the modelled osteotomies and the order in which the maps of the reduced stresses according to Huber are presented in the figures.

Model	Tooth-Borne Type Appliance	Bone-Borne Type Appliance
Model 1	No surgery	
Model 2	Palatal suture osteotomy	
Model 3	Le Fort I osteotomy without a PMJ separation	
Model 4	Le Fort I osteotomy with a PMJ separation	
Model 5	Palatal suture + Le Fort I osteotomy without a PMJ separation	
Model 6	Palatal suture + Le Fort I osteotomy with a PMJ separation	

PMJ—pterygomaxillary junction.

**Table 3:** Stress distribution (MPa) according to Huber throughout the craniofacial model with various surgical procedures and the tooth-borne and bone-borne orthodontic appliance using the finite element analysis.

Anatomical Structures	Tooth-Borne						Bone-Borne					
	No. 1	No. 2	No. 3	No. 4	No. 5	No. 6	No. 1	No. 2	No. 3	No. 4	No. 5	No. 6
Nasofrontal suture	2.2	5.5	1.1	1.1	4.4	2.2	3.3	5.5	3.3	2.2	5.5	2.2
Zygomaticomaxillary suture	3.3	5.5	4.4	1.1	4.4	2.2	>10	5.5	3.3	1.1	4.4	2.2
Arcus superciliaris—brow ridge	2.2	8.8	2.2	2.2	7.7	3.3	5.5	>10	5.5	3.3	10	3.3
Zygomaticofrontal suture	4.4	7.7	3.3	2.2	7.7	3.3	3.3	6.6	7.7	3.3	4.4	3.3
Palatal suture anterior region	>25	1.1	>25	>25	1.1	1.1	>25	1.1	>25	>25	1.1	1.1
Palatal suture posterior region	13.8	1.1	13.8	13.8	1.1	1.1	>25	1.1	>25	>25	1.1	1.1
Supraorbital margin	2.2	4.4	2.2	1.1	4.4	2.2	3.3	5.5	3.3	2.2	5.5	2.2
Infraorbital margin	4.4	5.5	2.2	1.1	7.7	2.2	>10	8.8	7.7	2.2	6.6	2.2
Apertura piriformis—the lowest point	5.5	1.1	>10	>10	1.1	1.1	>10	1.1	>10	>10	1.1	1.1
Anterior wall of the maxillary sinus	>10	4.4	7.7	1.1	3.3	2.2	>10	5.5	>10	2.2	7.7	3.3
Zygomaticoalveolar crest	>10	>10	5.5	3.3	5.5	3.3	>10	>10	>10	>10	5.5	3.3
Processus alveolaris of the maxillae regio incisors and canine	7.7	1.1	10	>10	1.1	1.1	>10	1.1	>10	>10	1.1	1.1
Processus alveolaris of the maxillae regio premolars	>10	8.8	>10	>10	7.7	3.3	>10	5.5	>10	>10	3.3	3.3
Processus alveolaris of the maxillae regio molars	5.5	1.1	5.5	7.7	3.3	3.3	>10	5.5	7.7	>10	3.3	2.2
Crown/Collum 5th maxilla tooth	>10	5.5	>10	>10	5.5	4.4	3.3	1.1	5.5	5.5	1.1	1.1

**Table 4:** Displacement values of selected anatomical structures (mm) along the *X*, *Y* and *Z*-axes, with various surgical procedures and the tooth-borne and bone-borne appliance using finite element analysis.

Axis	Variable	Tooth-Borne						Bone-Borne					
		No. 1	No. 2	No. 3	No. 4	No. 5	No. 6	No. 1	No. 2	No. 3	No. 4	No. 5	No. 6
*X*	Mesial incisal angle of maxillary tooth 1	+0.04	+0.31	+0.04	+0.04	+0.31	0.4	0.04	0.4	0.04	0.04	0.4	>0.4
	Buccal cusp tip of maxillary tooth 5	0.04	0.22	0.04	0.13	0.31	0.31	0.13	0.31	0.13	0.22	0.31	0.4
	Posterolateral surface of the maxilla	0.04	0.22	0.04	0.04	0.22	0.22	0.13	0.22	0.13	0.13	0.22	0.31
	Apertura piriformis—the lowest point	0.04	0.22	0.04	0.04	0.22	0.22	0.13	0.22	0.13	0.13	0.22	0.4
*Y*	Mesial incisal angle of maxillary tooth 1	−0.05	0.05	−0.05	−0.05	0.05	0.07	>−0.1	0.07	>−0.1	>−0.1	0.07	0.1
	Buccal cusp tip of maxillary tooth 5	0.01	0.03	0.01	0.03	0.03	0.01	0.01	0.05	0.03	0.07	0.05	0.01
	Posterolateral surface of the maxilla	0.01	0.03	0.03	0.05	0.03	−0.01	0.01	0.03	0.03	0.05	0.03	−0.03
	Apertura piriformis—the lowest point	−0.03	0.03	−0.03	−0.03	0.03	0.05	−0.05	0.05	−0.05	−0.03	0.03	0.05
*Z*	Mesial incisal angle of maxillary tooth 1	−0.03	0.01	−0.03	−0.03	−0.05	−0.05	−0.07	−0.05	−0.07	−0.03	−0.05	−0.03
	Buccal cusp tip of maxillary tooth 5	−0.03	0.07	0.01	0.03	0.07	0.05	0.01	0.07	0.03	0.1	0.05	0.05
	Posterolateral surface of the maxilla	0.03	0.07	0.03	0.07	0.07	0.07	0.03	0.07	0.05	>0.1	0.1	0.07
	Apertura piriformis—the lowest point	−0.03	0.01	−0.03	−0.03	−0.05	−0.05	−0.05	−0.03	−0.05	−0.05	−0.05	−0.05

*X*—buccolingual, (+)—buccal, (−)—lingual; *Y*—anterioposterior (front–back), (+)—anterior, (−)—posterior; *Z*—superioinferior (upper part–lower part), (+)—superior, (−)—inferior.

## Data Availability

The data presented in this study are available on request from the corresponding author.
